# Laparoscopic modified mesocolic excision with central vascular ligation in right-sided colon cancer shows better short- and long-term outcomes compared with the open approach in propensity score analysis

**DOI:** 10.1007/s00464-017-5970-6

**Published:** 2017-11-03

**Authors:** Jung Kyong Shin, Hee Cheol Kim, Woo Yong Lee, Seong Hyeon Yun, Yong Beom Cho, Jung Wook Huh, Yoon Ah Park, Ho-Kyung Chun

**Affiliations:** 1 Department of Surgery, Samsung Medical Center, Sungkyunkwan University School of Medicine, 81 Irwon-ro, Gangnam-gu, Seoul, 06351 South Korea; 2 Department of Surgery, Kangbuk Samsung Hospital, Sungkyunkwan University School of Medicine, Seoul, South Korea

**Keywords:** Modified complete mesocolic excision, Right-sided colon cancer, Laparoscopic

## Abstract

**Background:**

The introduction of complete mesocolic excision (CME) with central vessel ligation (CVL) for right-sided colon cancer has improved oncologic outcomes. However, there is controversy over the oncologic safety of laparoscopic CME with CVL. This study compared short-term and long-term oncologic outcomes between laparoscopic and open modified CME (mCME) with CVL in patients with right-sided colon cancer.

**Methods:**

We enrolled 1239 patients who underwent open mCME with CVL and 1010 patients treated by a laparoscopic approach for right-side colon cancer between 2000 and 2013 and used 1:1 propensity score matching to adjust for potential baseline confounders between two groups.

**Results:**

After propensity score matching, 683 patients who underwent open mCME with CVL were compared with 683 patients treated with a laparoscopic approach. There were no significant differences between these groups in age, sex, ASA score, TNM stage, tumor size, lymphovascular invasion, and perineural invasion. Comparison of open and laparoscopic mCME groups showed no significant difference in postoperative morbidity (21.4 vs. 18.3%, *p* = 0.175) and mortality (0.1 vs. 0%, *p* = 1.000). The laparoscopic mCME group showed shorter length of hospital stay. The 5-year overall survival rate was 83.7% in the open group and 94.7% in the laparoscopic group (*p* < 0.001). The laparoscopic group also showed a significantly better 5-year disease-free survival rate (82.7 vs. 88.7%, *p* = 0.009) and 5-year disease-specific survival rate (83.7 vs. 94.7%, *p* < 0.001).

**Conclusion:**

Laparoscopic modified mesocolic excision with central vascular ligation is a safe and feasible approach with better short-term recovery profiles and potential oncologic benefits than the open approach for right-sided colon cancer.

Total mesorectal excision (TME) is considered the standard technique for middle or lower rectal cancers. This surgical approach entails complete removal of the mesorectum circumferentially including all blood vessels, lymphatic vessels, and lymph nodes through which the tumor may disseminate [1, 2]. TME is currently applied worldwide, and has been shown to significantly increase disease-free survival rates and overall survival rates [3]. Likewise, the concept of complete mesocolic excision (CME) with central vascular ligation (CVL) in colon cancer surgery has been recently introduced. This surgical technique includes dissection of the mesocolon along the embryologic planes, resulting in complete mobilization of the mesocolon covered by an intact visceral fascia layer containing all blood vessels, lymphatic vessels, lymph nodes that may contain disseminated disease [4, 5]. In addition, ligation of the supplying vessels at their origin (CVL) and removal of the entire mesocolon has a significant effect on locoregional recurrence and improves oncologic outcomes [4–8]. A recent international consensus suggested that extended lymphadenectomy techniques should be the standard procedure for colon cancer patients [9–12]. In Japan, D3 lymphadenectomy is the standard method for stage II and stage III colon cancer and 5-year overall survival rates of up to 92% have been reported [13].

Recent data suggest that cancers of the right and left colon should be distinguished as they differ in clinical and molecular characteristics [14–17]. Many studies have reported that the oncologic outcomes are significantly poorer for right colon cancer than left colon cancer [17–20]. In clinical practice, it is more difficult to perform CME for right-sided colon cancer than left-sided colon cancer. Moreover, laparoscopic CME is not easy to perform because of the complex and variable vascular anatomy of the right colon. Several comparative studies between open and laparoscopic CME surgeries have been performed [21, 22]; however, these studies analyzed small numbers of patients and only a few reports long-term follow-up after laparoscopic CME. Furthermore, there are few studies comparing long-term outcomes between laparoscopic and open approaches specifically for right-sided colon cancers. Based on these embryologic, clinical, and molecular differences, our study focused on patients with right-sided colon cancer.

We have performed modified CME (mCME) using a surgical technique similar to the original approach of CME with CVL described by Hohenberger et al. [5] but with some technical differences for the treatment of right-sided colon cancer. Our modified CME right hemicolectomy technique is described in detail below.

The aim of this study was to compare the short-term and long-term outcomes of a large cohort undergoing modified CME with CVL by laparoscopic or open surgery for right-sided colon cancers.

## Patients and methods

All consecutive patients undergoing modified mesocolic excision with central vessel ligation for right-sided colon cancer at the Samsung Medical Center between January 2000 and December 2013 were analyzed. The right side of the colon was defined as the colon up to the middle transverse colon. During this period, 2249 patients with right-sided colon cancer underwent mCME with CVL. Of these, 1010 operations were performed by the laparoscopic approach and 1239 operations by an open procedure. Patients were designated for laparoscopic or open resection according to surgeon’s preference. The selection of the surgical approach is based on the surgeon’s discretion. However, there was a possibility that an open approach could be preferred when patients presented an advanced stage or a greater tumor size, especially in the early period of the present study. To decrease this bias, we conducted propensity score matching to adjust for potential baseline confounders between the groups.

For patients in the laparoscopic group, conventional or single-incision laparoscopic surgery was performed according to standard procedures. After propensity score matching a total of 1366 patients, 683 in the open group and 683 in the laparoscopy group were analyzed. Perioperative data, postoperative mortality and morbidity, and oncologic outcomes were compared between the laparoscopic and open groups.

All patients underwent preoperative chest and abdominopelvic computed tomography (CT). After discharge, the patients visited the outpatient clinic every 3 months for the first 2 years, every 6 months for the subsequent 3 years, and annually thereafter. Regular laboratory tests, chest CT, and abdominopelvic CT scans were performed every 6 months or every year during the follow-up period.

### Surgical technique

All modified CME surgeries were performed by seven colorectal surgeons with extensive open and laparoscopic surgical experience of more than 100 operations per year. They were equally experienced both in open and laparoscopic approaches, and experienced more than 100 cases of open as well as laparoscopic mCME before beginning of study.

A medial to lateral dissection was preferred in most cases. Similar to the original CME technique, modified CME was performed by sharp dissection between the visceral fascia and parietal fascia and ligation of the supplying vessels at their origin. However, several procedures differed from the original CME procedure described by Hohenberger et al. Unlike the original CME, we did not perform complete kocherization routinely in most cases unless the cancer cells had invaded the duodenum or perinephric fat tissue. If the tumor was locally advanced we dissected behind Gerota’s fascia, including perinephric fat tissues. With the exception of cases of transverse colon cancer, although we dissected the root of the midcolic artery and skeletonized it, we preserved the root and ligated only the right branch of the midcolic artery. After performing complete mobilization of the mesocolon, the supplying vessels were ligated to perform CVL. In cases of cecal or proximal ascending colon cancer, the ileocolic vessels and the right branch of the midcolic vessels were ligated at their origin from the superior mesenteric vessels. If the tumor was located at the hepatic flexure or proximal transverse colon, we ligated the midcolic vessels at their origin from the superior mesenteric vessels. The greater omentum was detached from the transverse colon for exposure of the lesser sac; however, unlike original CME, we did not perform ligation of all gastroepiploic vessels. The surgical technique for laparoscopic approach was performed under the same principle as for open surgery during the period.

### Statistical analysis

Analyses were performed using SPSS for Windows version 22.0 (SPSS, Chicago, IL, USA) and R2.15.3. Differences between two groups were analyzed using the Chi square test, Fisher’s exact test, or the Mann–Whitney *U* test, as appropriate. Survival rates were calculated by the Kaplan–Meier method, and survival curves were compared using the log-rank test. Results were considered statistically significant at *p* < 0.05.

Propensity matching was performed including the variables of age, sex, ASA score, pathologic stage, tumor size, adjuvant chemotherapy, lymphovascular invasion, perineural invasion, tumor budding, and intraoperative transfusion.

## Results

### Clinicopathologic characteristics of patients before and after propensity score matching

The clinicopathologic characteristics of patients included in this study are listed in Table 1. During the study period, mCME with CVL of right-sided colon cancer was performed in 1010 patients by laparoscopy and 1239 patients by the open approach. The median age was 62 years for the open group and 60 years for the laparoscopic group (*p* = 0.438). There were no significant differences between the groups in gender, body mass index, location of tumor, and adjuvant chemotherapy. Preoperative carcinoembryonic antigen (CEA) levels were significantly higher in the open group (9.21 vs. 5.02, *p* = 0.017).

**Table 1:** Demographic and pathologic data before and after propensity score matching

	Before propensity score matching			After propensity score matching		
	Open (*n* = 1239)	Laparoscopic (*n* = 1010)	*p*	Open (*n* = 683)	Laparoscopic (*n* = 683)	*p*
Age, years, median (SD)	62 ± 13	60 ± 11	0.438	61 ± 13	61 ± 12	0.565
BMI	23.2 ± 3.4	23.0 ± 2.6	0.088	23.2 ± 2.9	23.9 ± 2.9	0.773
Sex, n (%)			0.912			0.414
Male	543 (43.8)	445 (44.1)		372 (54.5)	388 (56.8)	
Female	696 (56.2)	565 (55.9)		311 (45.5)	295 (43.2)	
ASA score			0.755			0.631
1, 2	1207 (97.4)	986(97.6)		661 (96.8)	665 (97.4)	
3, 4	32 (2.6)	24(2.4)		22 (3.2)	18 (2.6)	
TNM stage			< 0.001			0.258
I	126 (10.2)	290 (28.7)		115 (16.8)	135 (19.8)	
II	603 (48.6)	370 (36.6)		305 (44.7)	280 (41.0)	
III	510 (41.2)	350 (34.7)		263 (38.5)	268 (39.2)	
Tumor size, mean ± SD, cm	6.2 ± 2.8	4.4 ± 3.0	< 0.001	5.1 ± 2.4	5.0 ± 2.6	0.318
Adjuvant CTx	759 (61.3)	428 (42.4)	< 0.001	343 (50.2)	340 (49.8)	0.914
Lymphatic invasion (+)	312 (25.2)	270 (26.7)	0.403	180(26.4)	180 (26.4)	1.000
Perineural invasion (+)	92 (7.4)	128 (12.7)	< 0.001	72 (10.5)	81 (11.9)	0.493
Vascular invasion (+)	134 (10.8)	91 (9.0)	0.156	70 (10.2)	59 (8.6)	0.355
Tumor budding (+)	226 (18.2)	382 (37.8)	< 0.001	206 (30.2)	206 (30.2)	1.000
Transfusion (+)	79 (6.4)	31 (3.1)	< 0.001	34 (5.0)	25 (3.7)	0.287

With respect to pathologic outcomes, the total number of retrieved lymph nodes was significantly greater in the open group (28.6 vs. 25.7, *p* = 0.005). However, no significant differences were noted between the two groups in terms of the number of positive lymph nodes and number of patients with fewer than 12 lymph nodes harvested.

The average tumor size (mean ± standard deviation) was 6.2 ± 2.8 cm in the open group and 4.4 ± 3.0 cm in the laparoscopic group (*p* < 0.001). The proximal resection margin (20.8 ± 13.5 vs. 15.1 ± 9.7 cm, *p* < 0.001) and distal resection margin (17.0 ± 9.1 vs. 15.2 ± 7.4, *p* < 0.001) were significantly greater in the open group compared with the laparoscopic group. However, in both groups, the proximal and distal resection margins were greater than 15 cm and there was no tumor involvement. There was no difference in radial margin between the two groups (5.2 ± 4.3 vs. 5.7 ± 4.1, *p* = 0.526). Perineural invasion was detected in 7.4% of the open group and 12.7% of the laparoscopic group, with a significant difference (*p* < 0.001). Lymphatic invasion and venous invasion were not significantly different between the two groups.

After matching, there were no significantly differences in any of the above factors between the two groups. The clinicopathologic characteristics of the matched patients are presented in Table 1.

### Perioperative and short-term outcomes

The perioperative and short-term outcomes for matched patients after the operation are shown in Table 2. The total operation time was significantly longer in the laparoscopic group than the open group (165 vs. 139 min, *p* < 0.001). The length of hospital stay was significantly shorter in the laparoscopic group (9.3 vs. 11.7 days, *p* < 0.001).

**Table 2:** Comparison of pathologic and short-term outcomes between the laparoscopy and open groups in matched cohorts

	Open (*n* = 683) (%)	Laparoscopic (*n* = 683) (%)	Total (*n* = 1366) (%)	*p*-value
Proximal margin				
Length, mean ± SD, cm	20.8 ± 13.5	15.1 ± 9.7	18.0 ± 12.1	< 0.001
Tumor involvement, n(%)	0 (0.0)	0 (0.0)	0 (0.0)	
Distal margin				
Length, mean ± SD, cm	17.0 ± 9.1	15.2 ± 7.4	16.1 ± 8.4	< 0.001
Tumor involvement, n (%)	0 (0.0)	0 (0.0)	0 (0.0)	
Radial margin				
Length, mean ± SD, mm	5.2 ± 4.3	5.7 ± 4.1	5.5 ± 4.2	0.526
Tumor involvement, n(%)	38 (3.1)	16 (1.6)	54 (2.4)	
Total no. retrieved LNs, mean ± SD	28.6 ± 13.9	25.7 ± 10.9	27.3 ± 12.8	0.005
No. of positive LNs, mean ± SD	1.5 ± 3.0	1.1 ± 2.4	1.3 ± 2.8	0.054
No. of cases with fewer than 12 LNs, n (%)	7 (0.6)	2 (0.2)	9 (0.4)	0.199
Total operation time (min)	139 ± 61	165 ± 50	152 ± 58	< 0.001
Hospital days	11.7 ± 4.7	9.3 ± 3.2	10.5 ± 4.2	< 0.001
Postop morbidity (+)	146 (21.4)	125 (18.3)		
Type of morbidity			271 (19.8)	0.175
Operation-related	114 (16.7)	102 (14.9)		0.335
Operation-unrelated	14 (2.0)	13 (1.9)		
Both	18 (2.6)	10 (1.5)		
Surgical site infection	50 (7.3)	11 (1.6)	61 (4.5)	< 0.001
Ileus	38 (5.6)	43 (6.3)	81 (5.9)	0.647
Anastomotic leakage	11 (1.6)	7 (1.0)	18 (1.3)	0.478
Intra-abdominal fluid collection	7 (1.0)	5 (0.7)	12 (0.9)	0.773
Intra-abdominal abscess	11 (1.6)	2 (0.3)	13 (1.0)	0.022
Intraluminal bleeding	4 (0.6)	6 (0.9)	10 (0.7)	0.753
Intra-abdominal bleeding	2 (0.3)	3 (0.4)	5 (0.4)	1.000
Postop mortality (< POD30)	1 (0.1)	0 (0.0)	1 (0.1)	1.000

Differences in overall postoperative morbidity rates between the groups did not reach significance; however, the rate of surgical site infection was significantly lower in the laparoscopic group (1.6 vs. 7.3%, *p* < 0.001). Details of short-term outcomes are shown in Table 3.

**Table 3:** Univariate and multivariate analysis of factors affecting postoperative morbidity

Factors	Univariate		Multivariate	
	HR (95% CI)	*p*	HR (95% CI)	*p*
Age (≥ 60 years)	1.275 (1.003–1.621)	0.048	1.510 (1.206–1.889)	< 0.001
Male	1.408 (1.117–1.774)	0.004	1.387 (1.112–1.730)	0.003
BMI (≥ 25)	1.054 (0.818–1.358)	0.682		
ASA score 3–4	1.898 (1.064–3.384)	0.030	3.048 (1.763–5.270)	< 0.001
Adjuvant CTx	1.692 (1.314–2.180)	< 0.001	1.588 (1.279–1.972)	< 0.001
Open approach	1.173 (0.931–1.480)	0.176		
Transfusion (+)	2.129 (1.364–3.323)	0.001	2.540 (1.688–3.822)	0.001
TNM stage III	1.453 (1.097–1.923)	0.009	1.252 (1.007–1.558)	0.044

*CTx* chemotherapy

Multivariate analysis for risk factors affecting postoperative complications was performed using the following variables: surgical approach (open vs. laparoscopy), age (< 60 vs. ≥60 years), sex, body mass index (< 25 vs. ≥ 25), ASA score (< 3 vs. ≥ 3), and harvested LNs (< 12 vs. ≥ 12). Age 60 years or older, male gender, ASA score 3 or 4, adjuvant chemotherapy, intraoperative transfusion, and advanced stage III disease were found to be independent prognostic factors (Table 3).

### Oncologic outcomes after propensity score matching

**Fig. 1:**
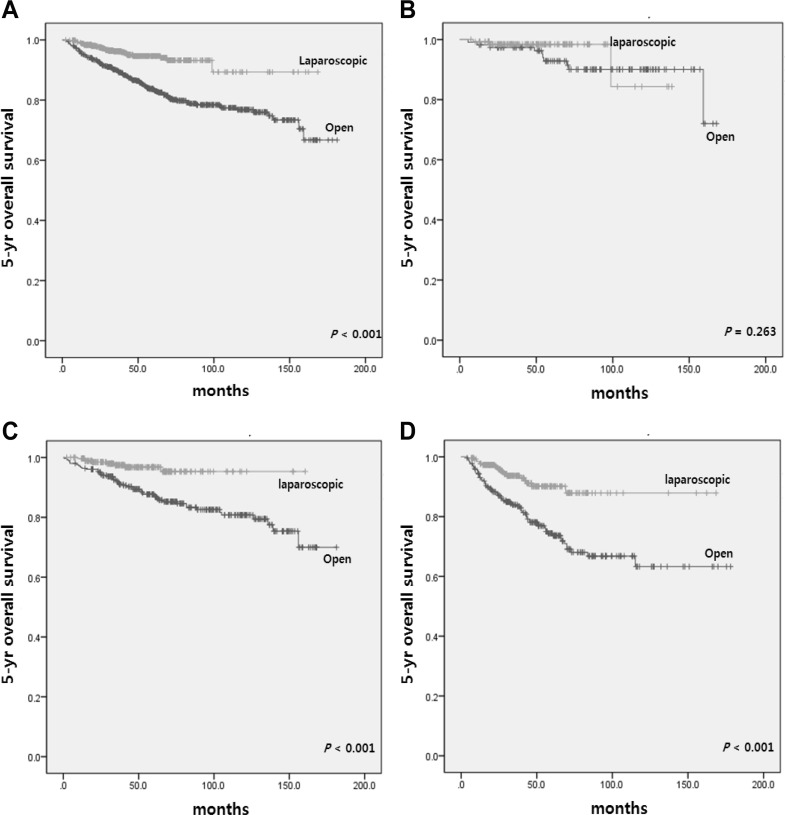
5-year overall survival according to operative approach in **A** all patients and patients with **B** stage I, **C** stage II, **D** stage III disease in matched cohorts

**Fig. 2:**
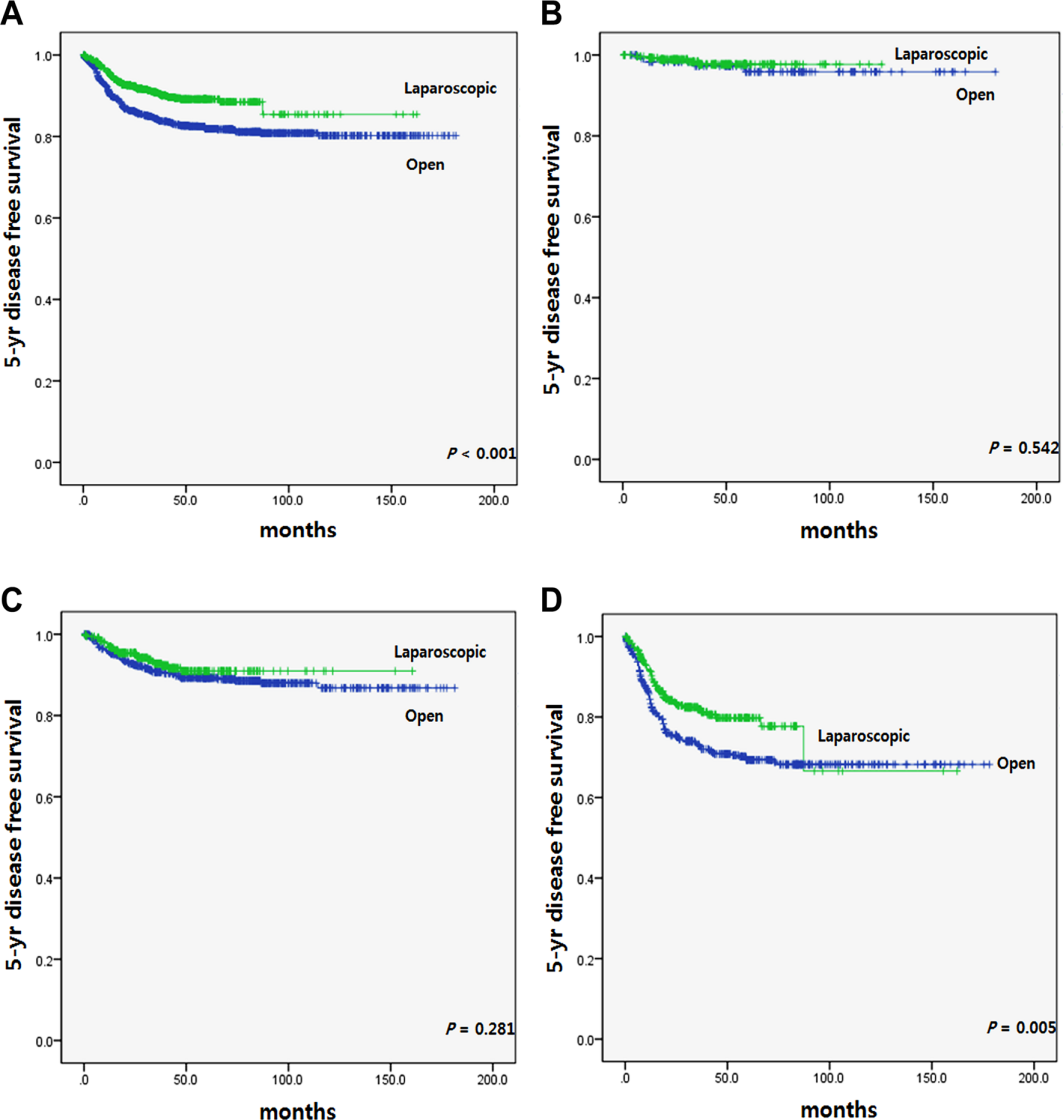
5-year disease-free survival according to operative approach in **A** all patients and patients with **B** stage I, **C** stage II, **D** stage III disease in matched cohorts

Survival analyses of matched patients are shown in Figs. 1, 2. The median follow-up period in the laparoscopic and open group was 41.0 and 55.1 months, respectively (*p* < 0.001).

The 5-year overall survival (OS) rate was significantly better for the laparoscopic group than the open group (94.7 vs. 83.7%, *p* < 0.001). Moreover, the laparoscopic modified CME group showed a higher 5-year OS rate for stage II and III disease than the open group (Fig. 1). The 5-year disease-free survival (DFS) rate in the laparoscopic group was also significantly higher than that in the open group (88.7 vs. 82.7%, *p* = 0.009) (Fig. 2).

Analysis of prognostic factors affecting 5-year OS is shown in Table 4. According to univariate analysis, factors associated with poorer overall survival were age 60 or older, adjuvant chemotherapy, open approach, TNM stage III, lymphatic invasion, and vascular invasion. Multivariate analysis identified age 60 or older, adjuvant chemotherapy, open approach, TNM stage III, and vascular invasion as independent prognostic factors.

**Table 4:** Univariate and multivariate analysis of factors affecting 5-year overall and disease-free survival

Factors	Overall survival			Disease-free survival		
	Univariate	Multivariate		Univariate	Multivariate	
	*p*	HR (95% CI)	*p*	*p*	HR (95% CI)	*p*
Age (≥ 60 years)	< 0.001	2.225 (1.723–2.873)	< 0.001	0.007	1.458 (1.149–1.852)	0.002
Male	0.378			0.205		
BMI (≥ 25)	0.289			0.515		
ASA score 3–4	0.617			0.373		
Adjuvant CTx	< 0.001	0.816 (0.648–1.027)	0.083	0.637		
Retrieved LNs ≥ 12	0.149			0.425		
Open approach	< 0.001	3.920 (2.754–5.580)	< 0.001	0.001	1.726 (1.344–2.216)	< 0.001
Transfusion (+)	0.382			0.039	2.280 (1.513–3.436)	< 0.001
TNM stage III	< 0.001	2.975 (2.355–3.757)	< 0.001	< 0.001	3.918 (3.064–5.012)	< 0.001
Lymphatic invasion	0.010	2.457 (1.385–4.360)		< 0.001	2.942 (2.334–3.708)	< 0.001
Perineural invasion	0.293			0.002	2.657 (1.965–3.593)	< 0.001
Vascular invasion	0.002	2.967 (2.237–3.935)	< 0.001	0.004	3.346 (2.537–4.412)	< 0.001

Analysis of prognostic factors affecting 5-year DFS is shown in Table 4. Age 60 or older, open approach, intraoperative transfusion, TNM stage III, and lymphatic, perineural, and vascular invasion were prognostic factors for 5-year DFS in the univariate analysis. All of the above factors remained as independent prognostic factors in the multivariate analysis.

### Patterns of recurrence

Among the 2249 patients with right-sided colon cancer who underwent curative primary tumor resection, 290 (12.9%) showed recurrence during the follow-up period (Table 5). Among all patients with tumor recurrence, 46 (2.0%) showed local recurrence, and 273 (12.1%) had a systemic recurrence. The most common sites of systemic recurrence were the liver (*n* = 81, 3.9%) and peritoneal seeding (*n* = 49, 2.2%), followed by para-aortic LN (*n* = 38, 1.8%), lung (*n* = 34, 1.6%), and ovary (*n* = 10, 0.5%). Between the two groups, the laparoscopy group showed less systemic recurrences during follow-up (*p* = 0.007, 15.0 vs. 8.3%). Local recurrence was observed in 37 (3.0%) patients in the open group and 9 (0.9%) patients in the laparoscopic group (*p* < 0.001). Locoregional tumor recurrences were mostly diagnosed at the anastomosis site (*n* = 33, 1.5%), abdominal wall or trocar site (*n* = 2, 0.1%), or common hepatic artery (*n* = 2, 0.1%). Only one patient who underwent open mCME had lymph node recurrence in peri-pancreatic region.

**Table 5:** Patterns of recurrences before and after propensity score matching

	Before propensity score matching			After propensity score matching		
	Open (*n* = 1239)	Laparoscopic (*n* = 1010)	*p*	Open (*n* = 683)	Laparoscopic (*n* = 683)	*p*
Systemic recurrence	186 (15.0)	84 (8.3)	< 0.001	95 (13.9)	60 (8.8)	0.004
Recurrence type			0.007			0.138
Liver	62 (5.0)	28 (2.8)		29 (4.2)	21 (3.1)	
Peritoneal seeding	32 (2.6)	16 (1.6)		13 (1.9)	13 (1.9)	
Paraaortic lymph node	31 (2.5)	12 (1.2)		20 (2.9)	9 (1.3)	
Lung	18 (1.5)	16 (1.6)		14 (2.0)	8 (1.2)	
Ovary	6 (0.5)	4 (0.4)		4 (0.6)	2 (0.3)	
Local recurrence	38 (3.1)	9 (0.9)	< 0.001	22 (3.2)	6 (0.9)	0.003
Recurrence type			0.004			0.002
Anastomosis site	26 (2.1)	4 (0.4)		16 (2.3)	1 (0.1)	
Trocar site	1 (0.1)	1 (0.1)		1 (0.1)	1 (0.1)	
Pancreas lymph node	1 (0.1)	0 (0.0)		1 (0.1)	0 (0.0)	

In matched cases, the systemic recurrences (*p* = 0.004, 13.9 vs. 8.8%) and local recurrences (*p* = 0.003, 3.2 vs. 0.9%) were higher in the open group.

## Discussion

Complete mesocolic excision involves resection of the tumor by sharp dissection of the visceral fascia from the parietal fascia layer together with the entire mesocolic fascia and surrounding soft tissue in an intact package. It removes hidden tumor deposits and avoids interruption of lymphatic and vascular drainage that may cause peritoneal cancer cell spillage. Although the term CME with CVL was first introduced by Hohenberger et al. [5], the procedure may not be a new one because similar concepts have previously been used by many surgeons. For instance, D3 lymphadenectomy, which is a similar technique to CME, is the standard care for clinical stage II and III colon cancer in Japan. Although the techniques share several similar concepts, there is some controversy regarding differences in surgical extent between D3 lymphadenectomy and CME with CVL. CME is a very aggressive surgical technique, and several surgeons have expressed concerns about an increased risk of postoperative morbidities.

In the present study, we performed modified CME with CVL for right-sided colon cancer. The results showed comparable pathologic results and long-term outcomes to those originally reported for CME by Hohenberger et al. [5]. As expected, overall and disease-free survival rates of the original CME data were comparable to those of other studies [5, 21–23]. Without removal of the retropancreatic lymph node and gastroepiploic lymph node, there was a rare recurrence in these areas in the present study, and the recurrence in these areas or lymph nodes has been rarely reported.

The median number of lymph nodes retrieved in our study was 27, which is smaller than that of Hohenberger et al. (median 32) [5] but equivalent to other CME studies [4, 5, 21, 23–27]. The R0 resection rate of 97.6% was identical to that of Hohenberger et al. (97.4%), and both studies excluded stage IV patients. The 5-year local recurrence rate for curative resection of 2.9% in this study was lower than that reported by Hohenberger et al. (4.9%) [5]. For curative resection, the 5-year overall survival rate of 86.9% was higher than the rates of 65.0% reported by West et al. [6] and 76.4% reported by the COST trial [28]; Hohenberger et al. did not report 5-year OS rates. The CLASSIC trial reported a 3-year OS of 68.4% [29]. This study showed a high 5-year disease-specific survival rate of 90.8 and 81.8% in all patients and those with stage III disease, respectively. These results were higher than those reported by Hohenberger et al. (90.8 vs. 85.0%) [5] and the Erlangen group (5-year DSS 89.0% for all stage and ≥ 90.0% for stage III patients) [30]. Furthermore, about the site of local recurrence, our results showed only one patient with regional recurrence in pancreatic lymph node. Our results of long-term oncologic outcomes indicate that mCME is an effective locoregional treatment for right-sided colon cancer, comparable to the original CME procedure.

We also compared the laparoscopic and open mCME groups before and after propensity score matching. The laparoscopic mCME was better than open mCME in terms of short- and long-term outcomes for right-sided colon cancer. However, although laparoscopic surgery may provide more favorable short-term outcomes, laparoscopic CME is difficult to perform because of the complex and variable vascular anatomy of the right colon. There are only a few reports on long-term follow-up after laparoscopic CME or comparing long-term outcomes between laparoscopic and open surgeries.

With respect to short-term outcomes, our study showed that laparoscopic mCME with CVL was both feasible and safe, with faster postoperative recovery than the open approach. Although the total operation time was significantly longer in the laparoscopic group, there was no significant difference in overall postoperative morbidity. Moreover, surgical site infection rate was significantly lower in the laparoscopic group compared with the open group.

The laparoscopic mCME specimens were comparable to those of open mCME in amount and quality in terms of proximal margin, distal margin, and radial margin. Recently, some studies have reported the importance of retrieved lymph node number after colon cancer surgery for oncologic outcome and have shown that a higher lymph node yield is associated with higher survival rates [8, 31]. The median number of lymph nodes harvested was 26 and 29 in the laparoscopic and open groups, respectively (*p* = 0.005). There were several possible explanations for this difference: in some cases of open surgery, more meticulous lymph node dissection could be performed. When comparing patient demographics, an open approach was preferred if the tumor is locally advanced and staged as T4a or T4b, or for a greater tumor size. In the advanced stage, we might have a more aggressive dissection of the lymph nodes over the head of the pancreas or along the gastroepiploic arcade. However, the difference in the number of harvested lymph nodes between the two groups might be related to the total length of the specimen. In a comparative study [30], the lymph node number was associated with the length of specimen without a difference in oncologic outcomes. In previous studies, the mean number of harvested lymph nodes after right-sided colon cancer surgery ranged from 15 to 35 for open CME and from 19 to 34 for laparoscopic CME [4, 5, 21, 23–27]. Although open mCME allows removal of more lymph nodes than laparoscopic mCME, both the open and laparoscopic approaches satisfy the current recommendation of a minimum of 12 lymph nodes [32]. The clinical significance of greater nodal clearance by open mCME should be studied further. Although in our study laparoscopic mCME showed significantly smaller proximal and distal margins than the open approach, both groups satisfied the traditional 5 or 10-cm rule for proximal and distal margins [5, 30, 33, 34]. Our findings agreed with previous results showing that laparoscopic CME is feasible and safe with respect to pathologic outcomes [25–27, 35].

Regarding long-term oncologic outcomes, laparoscopic mCME showed significantly better results than open mCME in our study. The multivariate analysis showed that an open surgical approach was an independent risk factor for decreased 5-year overall and disease-free survival rates. There are several suggestions for the better oncologic outcomes in the laparoscopic group. First, of course, there was a selection bias because laparoscopic surgery was more frequently performed in patients with early-stage tumors. In the present study, more T4 cases were involved in the open group, even after propensity matching including the stage. This mismatching of T4 cancer seemed to be one of the reasons for the poor oncologic outcomes in the open group. However, in the sub-group analysis for only T4 cancer between the two groups, three was a difference regarding the OS and no difference regarding the DFS and LRFS. Based on our data, even for T4 cancers in the right side colon, a laparoscopic approach might give better oncologic outcomes. The mechanism of this phenomenon may be the subject of further research. Second, another possible statistical confounding factor may be the shorter follow-up period in the laparoscopic group. Third, many laparoscopic cases were conducted in a relatively recent period compared to the open group. Despite the lack of a difference in chemotherapy between the two groups, improved chemotherapy regimens and improvements in the operative technique could have contributed to reduced local recurrence rates in patients who underwent laparoscopic resection. Finally, there is a possibility of the laparoscopic technique itself providing oncologic benefits, including less handling of the tumor and better immune function after surgery.

There are selected reports on long-term outcomes of laparoscopic CME for right-sided colon cancer, but this study is the one of the largest cohort studies comparing long-term follow-up results of laparoscopic CME vs. open CME for right-sided colon cancer. Takatoshi et al. performed a case-matched comparison between laparoscopic and open right hemicolectomy with excellent long-term oncologic outcomes in the laparoscopic group [36]. There are also nonrandomized comparative studies that have shown better long-term oncologic outcomes in the laparoscopic group compared with the open group [27, 37, 38]. In contrast, Bae et al. reported comparable long-term oncologic outcomes between the laparoscopic and open groups [21] and several studies have not shown any significant difference in survival between the two groups [28, 36, 39–41].

Cho et al. [27] reported that both 5-year OS and 5-year DSS rates were significantly better in the minimally invasive surgery group than in the open group (89.8 vs. 82.4%, *p* = 0.023; 90.8 vs. 84.2%, *p* = 0.015, respectively). However, they did not analyze demographics such as age, sex, ASA score, BMI, and TNM stage in each group. These factors might have influenced postoperative outcomes, including morbidity and oncologic outcomes. In this study, age, sex, BMI, and ASA score 3 or 4 did not differ significantly between the two groups. Furthermore, when compared overall survival rates between the laparoscopic and open mCME groups, the laparoscopic group showed better oncologic outcomes when stratified by TNM stages. Bae et al. also showed a better overall survival rate in the laparoscopic group, but their analysis did not include stratification by TNM stages [21]. The benefits of a laparoscopic approach include a significantly better postoperative outcome that allows a shorter hospital stay with a faster return to normal life. These factors may positively influence the oncologic outcome, allowing early treatment with adjuvant chemotherapy, better acceptance of repeated operations for recurrence, and preservation of immune function.

There were several limitations in this study. First, this was a retrospective study with a single-center design. Second, the follow-up period was shorter in the laparoscopic group. This was mainly because most of the laparoscopic procedures were performed in the later part of the study, and this difference may have influenced recurrence rates as well as long-term overall and disease-free survival. Despite these limitations, we analyzed a large number of patients who underwent laparoscopic modified complete mesocolic excision.

In conclusion, modified complete mesocolic excision is comparable to the original CME procedure in terms of short- and long-term outcomes. Laparoscopic mCME for right-sided colon cancer showed short-term benefits in terms of faster recovery and reduced surgical site infection. Moreover, laparoscopic mCME demonstrated better oncologic outcomes than open mCME. This study suggested that laparoscopic mCME is a safe and effective procedure for right-sided colon cancer with potential oncologic benefits.

## References

[1] Heald RJ, Husband EM, Ryall RD (1982). The mesorectum in rectal cancer surgery–the clue to pelvic recurrence?. Br J Surg.

[2] Heald RJ, Ryall RD (1986). Recurrence and survival after total mesorectal excision for rectal cancer. Lancet.

[3] Quirke P, Steele R, Monson J, Grieve R, Khanna S, Couture J, O’Callaghan C, Myint AS, Bessell E, Thompson LC, Parmar M, Stephens RJ, Sebag-Montefiore D (2009). Effect of the plane of surgery achieved on local recurrence in patients with operable rectal cancer: a prospective study using data from the MRC CR07 and NCIC-CTG CO16 randomised clinical trial. Lancet.

[4] Gouvas N, Pechlivanides G, Zervakis N, Kafousi M, Xynos E (2012). Complete mesocolic excision in colon cancer surgery: a comparison between open and laparoscopic approach. Colorectal Dis.

[5] Hohenberger W, Weber K, Matzel K, Papadopoulos T, Merkel S (2009). Standardized surgery for colonic cancer: complete mesocolic excision and central ligation–technical notes and outcome. Colorectal Dis.

[6] West NP, Morris EJ, Rotimi O, Cairns A, Finan PJ, Quirke P (2008). Pathology grading of colon cancer surgical resection and its association with survival: a retrospective observational study. Lancet Oncol.

[7] West NP, Hohenberger W, Weber K, Perrakis A, Finan PJ, Quirke P (2010). Complete mesocolic excision with central vascular ligation produces an oncologically superior specimen compared with standard surgery for carcinoma of the colon. J Clin Oncol.

[8] Le Voyer TE, Sigurdson ER, Hanlon AL, Mayer RJ, Macdonald JS, Catalano PJ, Haller DG (2003). Colon cancer survival is associated with increasing number of lymph nodes analyzed: a secondary survey of intergroup trial INT-0089. J Clin Oncol.

[9] Sondenaa K, Quirke P, Hohenberger W, Sugihara K, Kobayashi H, Kessler H, Brown G, Tudyka V, D’Hoore A, Kennedy RH, West NP, Kim SH, Heald R, Storli KE, Nesbakken A, Moran B (2014). The rationale behind complete mesocolic excision (CME) and a central vascular ligation for colon cancer in sopen and laparoscopic surgery: proceedings of a consensus conference. Int J Colorectal Dis.

[10] Storli KE, Sondenaa K, Furnes B, Nesvik I, Gudlaugsson E, Bukholm I, Eide GE (2014). Short term results of complete (D3) vs. standard (D2) mesenteric excision in colon cancer shows improved outcome of complete mesenteric excision in patients with TNM stages I-II. Tech Coloproctol.

[11] Bertelsen CA, Neuenschwander AU, Jansen JE, Wilhelmsen M, Kirkegaard-Klitbo A, Tenma JR, Bols B, Ingeholm P, Rasmussen LA, Jepsen LV, Iversen ER, Kristensen B, Gogenur I, Danish Colorectal Cancer Group (2015). Disease-free survival after complete mesocolic excision compared with conventional colon cancer surgery: a retrospective, population-based study. Lancet Oncol.

[12] Rosenberg R, Engel J, Bruns C, Heitland W, Hermes N, Jauch KW, Kopp R, Putterich E, Ruppert R, Schuster T, Friess H, Holzel D (2010). The prognostic value of lymph node ratio in a population-based collective of colorectal cancer patients. Ann Surg.

[13] Yamamoto S, Inomata M, Katayama H, Mizusawa J, Etoh T, Konishi F, Sugihara K, Watanabe M, Moriya Y, Kitano S (2014). Short-term surgical outcomes from a randomized controlled trial to evaluate laparoscopic and open D3 dissection for stage II/III colon cancer: Japan Clinical Oncology Group Study JCOG 0404. Ann Surg.

[14] Bufill JA (1990). Colorectal cancer: evidence for distinct genetic categories based on proximal or distal tumor location. Ann Intern Med.

[15] Price TJ, Beeke C, Ullah S, Padbury R, Maddern G, Roder D, Townsend AR, Moore J, Roy A, Tomita Y, Karapetis C (2015). Does the primary site of colorectal cancer impact outcomes for patients with metastatic disease?. Cancer.

[16] Benedix F, Meyer F, Kube R, Gastinger I, Lippert H (2010). Right- and left-sided colonic cancer—different tumour entities. Zentralbl Chir.

[17] Benedix F, Kube R, Meyer F, Schmidt U, Gastinger I, Lippert H (2010). Comparison of 17,641 patients with right- and left-sided colon cancer: differences in epidemiology, perioperative course, histology, and survival. Dis Colon Rectum.

[18] Nitsche U, Stogbauer F, Spath C, Haller B, Wilhelm D, Friess H, Bader FG (2016). Right sided colon cancer as a distinct histopathological subtype with reduced prognosis. Dig Surg.

[19] Meguid RA, Slidell MB, Wolfgang CL, Chang DC, Ahuja N (2008). Is there a difference in survival between right- versus left-sided colon cancers?. Ann Surg Oncol.

[20] Verhulst J, Ferdinande L, Demetter P, Ceelen W (2012). Mucinous subtype as prognostic factor in colorectal cancer: a systematic review and meta-analysis. J Clin Pathol.

[21] Bae SU, Saklani AP, Lim DR, Kim DW, Hur H, Min BS, Baik SH, Lee KY, Kim NK (2014). Laparoscopic-assisted versus open complete mesocolic excision and central vascular ligation for right-sided colon cancer. Ann Surg Oncol.

[22] Huang JL, Wei HB, Fang JF, Zheng ZH, Chen TF, Wei B, Huang Y, Liu JP (2015). Comparison of laparoscopic versus open complete mesocolic excision for right colon cancer. Int J Surg.

[23] Shin JW, Amar AH, Kim SH, Kwak JM, Baek SJ, Cho JS, Kim J (2014). Complete mesocolic excision with D3 lymph node dissection in laparoscopic colectomy for stages II and III colon cancer: long-term oncologic outcomes in 168 patients. Tech Coloproctol.

[24] Bokey L, Chapuis PH, Chan C, Stewart P, Rickard MJ, Keshava A, Dent OF (2016). Long-term results following an anatomically based surgical technique for resection of colon cancer: a comparison with results from complete mesocolic excision. Colorectal Dis.

[25] Adamina M, Manwaring ML, Park KJ, Delaney CP (2012). Laparoscopic complete mesocolic excision for right colon cancer. Surg Endosc.

[26] Siani LM, Garulli G (2016). Laparoscopic complete mesocolic excision with central vascular ligation in right colon cancer: a comprehensive review. World J Gastrointest Surg.

[27] Kim YI, Kim YW, Choi IJ, Kim CG, Lee JY, Cho SJ, Eom BW, Yoon HM, Ryu KW, Kook MC (2015). Long-term survival after endoscopic resection versus surgery in early gastric cancers. Endoscopy.

[28] Fleshman J, Sargent DJ, Green E, Anvari M, Stryker SJ, Beart RW, Hellinger M, Flanagan R, Peters W, Nelson H (2007). Laparoscopic colectomy for cancer is not inferior to open surgery based on 5-year data from the COST Study Group trial. Ann Surg.

[29] Jayne DG, Guillou PJ, Thorpe H, Quirke P, Copeland J, Smith AM, Heath RM, Brown JM, Group UMCT (2007). Randomized trial of laparoscopic-assisted resection of colorectal carcinoma: 3-year results of the UK MRC CLASICC Trial Group. J Clin Oncol.

[30] West NP, Kobayashi H, Takahashi K, Perrakis A, Weber K, Hohenberger W, Sugihara K, Quirke P (2012). Understanding optimal colonic cancer surgery: comparison of Japanese D3 resection and European complete mesocolic excision with central vascular ligation. J Clin Oncol.

[31] Chang GJ, Rodriguez-Bigas MA, Skibber JM, Moyer VA (2007). Lymph node evaluation and survival after curative resection of colon cancer: systematic review. J Natl Cancer Inst.

[32] Provenzale D, Jasperson K, Ahnen DJ, Aslanian H, Bray T, Cannon JA, David DS, Early DS, Erwin D, Ford JM, Giardiello FM, Gupta S, Halverson AL, Hamilton SR, Hampel H, Ismail MK, Klapman JB, Larson DW, Lazenby AJ, Lynch PM, Mayer RJ, Ness RM, Rao MS, Regenbogen SE, Shike M, Steinbach G, Weinberg D, Dwyer MA, Freedman-Cass DA, Darlow S (2015). Colorectal cancer screening, version 1.2015. J Natl Compr Canc Netw.

[33] Okuno K (2007). Surgical treatment for digestive cancer. Current issues—colon cancer. Dig Surg.

[34] Watanabe T, Itabashi M, Shimada Y, Tanaka S, Ito Y, Ajioka Y, Hamaguchi T, Hyodo I, Igarashi M, Ishida H, Ishihara S, Ishiguro M, Kanemitsu Y, Kokudo N, Muro K, Ochiai A, Oguchi M, Ohkura Y, Saito Y, Sakai Y, Ueno H, Yoshino T, Boku N, Fujimori T, Koinuma N, Morita T, Nishimura G, Sakata Y, Takahashi K, Tsuruta O, Yamaguchi T, Yoshida M, Yamaguchi N, Kotake K, Sugihara K, Japanese Society for Cancer of the Colon and Rectum (2015). Japanese Society for Cancer of the Colon and Rectum (JSCCR) Guidelines 2014 for treatment of colorectal cancer. Int J Clin Oncol.

[35] Kim IY, Kim BR, Choi EH, Kim YW (2016). Short-term and oncologic outcomes of laparoscopic and open complete mesocolic excision and central ligation. Int J Surg.

[36] Nakamura T, Onozato W, Mitomi H, Naito M, Sato T, Ozawa H, Hatate K, Ihara A, Watanabe M (2009). Retrospective, matched case-control study comparing the oncologic outcomes between laparoscopic surgery and open surgery in patients with right-sided colon cancer. Surg Today.

[37] Bilimoria KY, Bentrem DJ, Nelson H, Stryker SJ, Stewart AK, Soper NJ, Russell TR, Ko CY (2008). Use and outcomes of laparoscopic-assisted colectomy for cancer in the United States. Arch Surg.

[38] Guerrieri M, Campagnacci R, De Sanctis A, Lezoche G, Massucco P, Summa M, Gesuita R, Capussotti L, Spinoglio G, Lezoche E (2012). Laparoscopic versus open colectomy for TNM stage III colon cancer: results of a prospective multicenter study in Italy. Surg Today.

[39] Li JC, Leung KL, Ng SS, Liu SY, Lee JF, Hon SS (2012). Laparoscopic-assisted versus open resection of right-sided colonic cancer—a prospective randomized controlled trial. Int J Colorectal Dis.

[40] Sun YW, Chi P, Lin HM, Lu XR, Huang Y, Xu ZB, Huang SH (2012). [Comparison of efficacy between laparoscopic versus open complete mesocolic excision for colon cancer]. Zhonghua Wei Chang Wai Ke Za Zhi.

[41] Athanasiou CD, Markides GA, Kotb A, Jia X, Gonsalves S, Miskovic D (2016). Open compared with laparoscopic complete mesocolic excision with central lymphadenectomy for colon cancer: a systematic review and meta-analysis. Colorectal Dis.

